# Remarks on Mr. Murphy's Natural History of the Human Teeth

**Published:** 1814-04

**Authors:** 


*For the Medical and Physical Journal.*

### *Remarks on Mr. Murphy's Natural History of the Human Teeth.*

WHEN first I saw notice for the publication of a Natural History of the Human Teeth, (after the scientific work written by Mr. Fox on that subject,) I was naturally led to suppose the author had gleaned some very useful information from his practice, or made some material improvements in the operations which are performed on them: this induced me to procure the book, and peruse it with attention. How far my expectations were realized, the remarks which are subjoined will determine.

The author observes in the beginning, and also repeats in various parts of his work, how much the beauty of the face depends upon the regular growth and formation of the teeth: he is even in doubt whether the loss of an eye or a front tooth tooth is the greatest deformity. This I thought was nothing but a fair sort of manœuvre to establish the necessity of consulting the dentist; for if we can persuade people that personal qualifications can be gained, however vain or ridiculous the means may appear, they will seize them with avidity; and if the loss of a tooth be equivalent in deformity to that of an eye, of how great importance is it that the dentist should be resorted to, by whose art all these defects can be remedied!

Proceeding forwards, we have an anatomical descriptio of the bones composing the orbit of the eye, nose, as well as the jaws; also the formation of the teeth, their number, order of cutting, &c. The whole, with the least possible alteration, may be found in a system of anatomy now very generally used in the schools. In this it may be said, how could you expect to find any thing novel, since the subject has been so ably elucidated by Mr. Hunter and others? I agree, very little; but the copying might have been more disguised, and if copying was at all necessary, why not do it from the best authors on the subject?

Speaking of epulis or gum-boil, he tells us, that when the effects of the disease have extended to the vessels at the point of the fang, and to the periosteum and socket, the circulation being *impeded* in those parts induces inflammation; suppuration takes place. Going on further, when the matter is formed, it *erodes* the socket on the outer side opposite to where it had formed, and bursts through the gum. How the want of circulation can produce inflammation, I am totally at a loss to conceive, as the general doctrine of inflammation has for one of its characteristics an *increase* of circulation, on which depend the redness and heat that is produced. When the matter is formed, it is said to *erode* the socket! This appears to be very curious, as it seems to erode its way to the surface. Now if it was by any acrid quality which the matter possessed, that we were to account for the bursting of an abscess; I would beg to ask how it is that the contents, however situated, have such an universal tendency to make their way to the surface of the body, or some cavity into which they can be discharged? It would be fair to suppose that matter would make its way according to gravitation, for there its greatest surface would be in contact with the containing parts; but that this does not happen, every day's experience must show. The matter contained in the gum-boil, after it has eroded the socket, bursts through the gum. Now, when the business of eating through the alveolar process has been accomplished, it no longer erodes, but forces its way. Perhaps from the action between it it and the bone, some gasses may have been formed which have the forcing or bursting power. This does not seem to agree at all with our author's opinion of the manner of cutting the teeth, when he condemns the practice of giving hard substances into the mouths of children to facilitate that operation, for there, he says, it is not a mechanical process, but proceeds from the absorption of the gum; and I should be inclined to say, may not absorption be the means by which the matter of gum-boil makes its way out? Was it to be said diseases of the teeth had been entirely forgot, it might be liable to contradiction. Some cures for the tooth-ache are certainly put down; and here the necessity of consulting the dentist is again hinted at, as we are told, it is now customary in polite seminaries to have the regular attendance of a dentist, he being capable, with the assistance of a magnifying mirror, to discover the decay long before any other person.

Was it not trespassing on your valuable pages, much might have been advanced to show (in the present instance at least) how little the minute divisions of surgery have conduced to its advancement. When a man fixes his whole attention to the diseases of one particular part, as the teeth, eye, &c. &c. it generally happens his mind becomes contracted in proportion to his attention: on the contrary, when a man has fully studied the anatomy, physiology, and diseases of the whole body, and fixes on one particular part, his collateral knowledge is like a light shining from many points to illumine his subject.

The object I have in writing this is not from any personal hostility to the author, but merely to call the attention of medical men to the system of book-making, which has become so very prevalent, and injurious in its consequences.

